# The prognostic role of single cell invasion and nuclear diameter in early oral tongue squamous cell carcinoma

**DOI:** 10.1186/s12885-024-11954-y

**Published:** 2024-02-15

**Authors:** Alhadi Almangush, Jaana Hagström, Caj Haglund, Luiz Paulo Kowalski, Ricardo D. Coletta, Antti A. Mäkitie, Tuula Salo, Ilmo Leivo

**Affiliations:** 1 Department of Pathology, University of Helsinki, FI-00014 Helsinki, Haartmaninkatu, P.O. Box 21, Finland; 2 Institute of Biomedicine, Pathology, University of Turku, Turku, Finland; 3 Research Program in Systems Oncology, Faculty of Medicine, University of Helsinki, Helsinki, Finland; 4 Faculty of Dentistry, Misurata University, Misurata, Libya; 5 Research Programs Unit, Translational Cancer Medicine, University of Helsinki, 00014 Helsinki, P.O. Box 63, Finland; 6 Department of Oral Pathology and Radiology, University of Turku, Turku, Finland; 7 Department of Surgery, University of Helsinki and Helsinki University Hospital, Helsinki, Finland; 8 Department of Head and Neck Surgery and Otorhinolaryngology, A.C. Camargo Cancer Center, Department of Head and Neck Surgery, University of Sao Paulo Medical School, 05402-000 São Paulo, SP Brazil; 9 Department of Oral Diagnosis and Graduate Program in Oral Biology, School of Dentistry, University of Campinas, 13414-018 Piracicaba, São Paulo, Brazil; 10 Department of Otorhinolaryngology– Head and Neck Surgery, University of Helsinki and Helsinki University Hospital, FI-00029 HUS Helsinki, P.O. Box 263, Finland; 11 Division of Ear, Nose and Throat Diseases, Department of Clinical Sciences, Intervention and Technology, Karolinska Institutet and Karolinska University Hospital, Stockholm, Sweden; 12 Department of Oral and Maxillofacial Diseases, University of Helsinki, Helsinki, Finland; 13 Institute of Biomedicine, Pathology, University of Turku, Turku University Central Hospital, 20520 Turku, Finland

**Keywords:** Oral tongue cancer, Single cell invasion, Large nuclei, Early stage, Survival

## Abstract

**Background:**

The clinical significance of single cell invasion and large nuclear diameter is not well documented in early-stage oral tongue squamous cell carcinoma (OTSCC).

**Methods:**

We used hematoxylin and eosin-stained sections to evaluate the presence of single cell invasion and large nuclei in a multicenter cohort of 311 cases treated for early-stage OTSCC.

**Results:**

Single cell invasion was associated in multivariable analysis with poor disease-specific survival (DSS) with a hazard ratio (HR) of 2.089 (95% CI 1.224–3.566, *P* = 0.007), as well as with disease-free survival (DFS) with a HR of 1.666 (95% CI 1.080–2.571, *P* = 0.021). Furthermore, large nuclei were associated with worse DSS (HR 2.070, 95% CI 1.216–3.523, *P* = 0.007) and with DFS in multivariable analysis (HR 1.645, 95% CI 1.067–2.538, *P* = 0.024).

**Conclusion:**

Single cell invasion and large nuclei can be utilized for classifying early OTSCC into risk groups.

## Background

The incidence of oral tongue squamous cell carcinoma (OTSCC) has increased in many regions including the Western countries. This tendency occurs also in the young age group [1, 2]. Furthermore, there are many patients with early OTSCC who have developed locoregional recurrence and/or died due to cancer-related mortality [3, 4]. Therefore, OTSCC still forms a major health burden in many societies. In daily practice of pathology and following the criteria of World Health Organization (WHO) classification, OTSCC is graded according to the degree of tumor keratinization and cell differentiation into well-, moderately- and poorly-differentiated tumors [5]. Unfortunately, this time-honoured grading system has shown limited prognostic value, also in early OTSCC [3, 6–8]. Thus, search for clinically relevant and reliable histopathologic prognostic classifiers that can be implemented in daily practice is necessary.

Hallmarks of cancer include active invasive growth into adjacent tissues [9]. Invasive growth plays critical role in determining an individual tumor’s clinical behavior [10]. Single cell invasion, defined as detachment of single cancer cells from the main tumor mass usually in the invasive front area, is one characteristic of aggressive growth that has been studied in different tumor types [11, 12]. In early OTSCC, however, the significance of single cell invasion has not been well elucidated.

Evaluation of the nuclear abnormalities including increased nuclear size was introduced long time ago as a criterion for malignancy in different tumor types [13–15]. In addition, evaluation of the nuclear disorder and characteristics has proven to carry important prognostic value [16]. Notably, studies that assessed nuclear diameter confirmed that large nuclei with a diameter greater than four small lymphocytes are associated with poor prognosis [11, 17, 18]. In most of the published studies, the evaluation of nuclear morphology was performed using routine hematoxylin and eosin (HE)-stained Sections. [11, 17, 19]. However, the clinical significance of increased nuclear diameter has not been well studied in early-stage OTSCC.

**Fig. 1:**
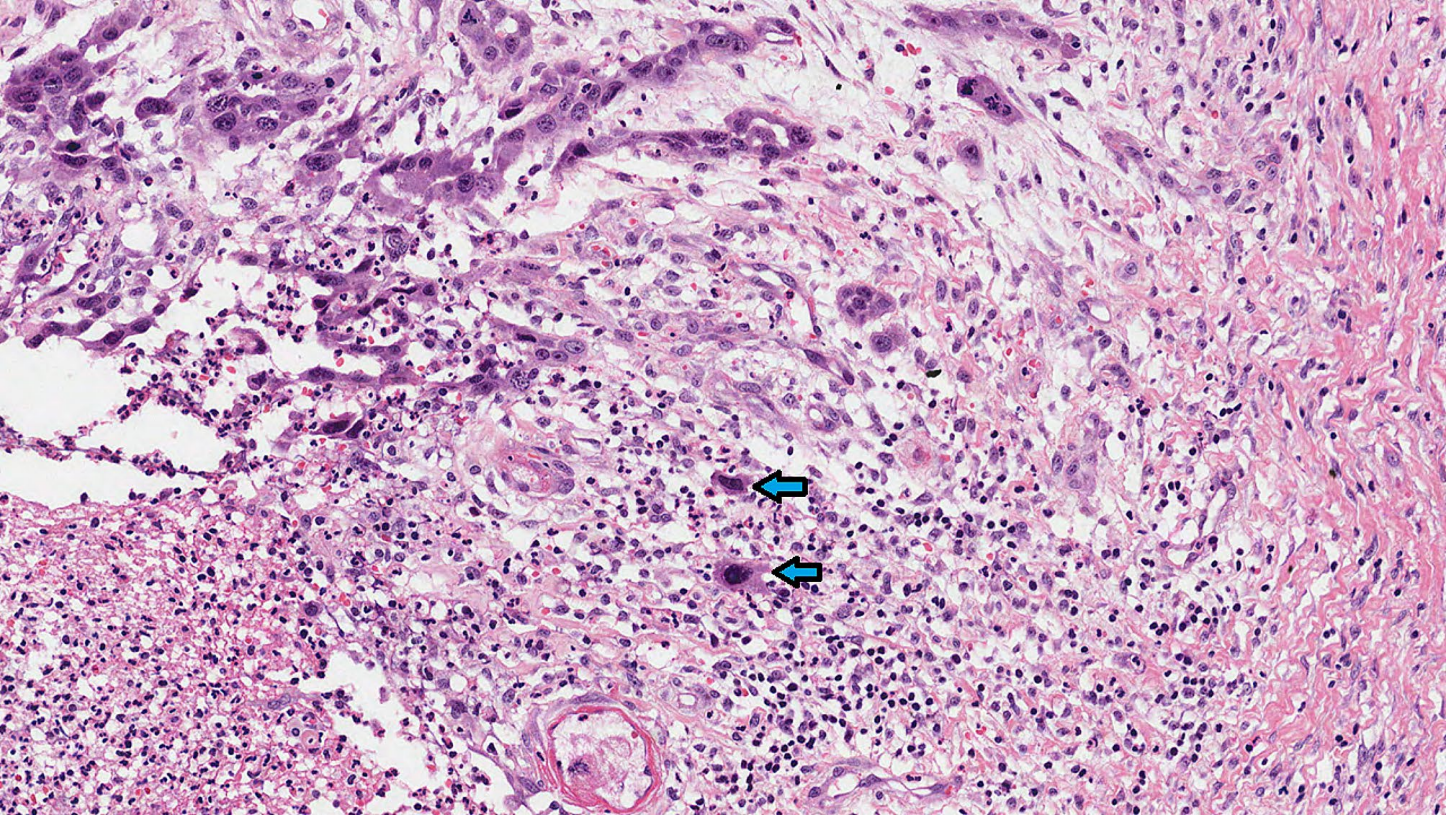
Hematoxylin and eosin-stained section of early-stage oral tongue cancer (magnification ×200). Single cell invasion presented at the same time with large nuclei (arrows). Single cell invasion refers to detachment of single cancer cells from the main tumor mass, and large nuclei are greater in diameter than four nearby small lymphocytes

In this multicenter study, we aimed to evaluate the prognostic significance of single cell invasion and large nuclei in early-stage OTSCC (Fig. 1) using routine HE-stained sections.

## Methods

A total of 311 cases treated for early (T1-T2N0M0) OTSCC between 1979 and 2012 at one of the five Finnish university hospitals (Helsinki, Turku, Tampere, Oulu, Kuopio) or at the A.C. Camargo Cancer Center, São Paulo, Brazil were included in this study. All included cases were clinically at an early stage (cT1-2N0), and they were treated with surgical resection. Permissions of the ethical committees of the included hospitals as well as permissions of the National Supervisory Authority for Welfare and Health in Finland and the Brazilian Human Research Ethics Committee were obtained.

To assess single cell invasion, we visually scanned entire tumor resection sections at intermediate magnification (×10 objective) and then assessed single cell invasion at higher magnification (×20 and ×40 objectives) at the particular area of the invasive front where the maximal number of the smallest clusters of cancer cells are found as previously reported [17]. For the evaluation of nuclear diameter, large nuclei were defined as being larger than the sum of the diameters of four small nearby lymphocytes as described in previous studies [11]. This differentiates them from small nuclei the largest diameter of which is less than the sum of the diameters of four small nearby lymphocytes. In addition, using diagnostic histopathological criteria we ensured that only cancer cells with large nuclei were evaluated, and not macrophages.

Two observers (AA & IL) arranged a training session for scoring of single cell invasion and large nuclei. The observers were unaware of the clinical data. We arranged a review consensus session to re-assess all cases which had a disagreement between the observers. We were able to reach consensus scores for such cases.

### Statistical method

We used SPSS 27 for all statistical analyses. We reported hazard ratio (HR) and 95% confidence interval (CI) for both univariable and multivariable analyses. A *P* value of less than 0.05 was considered a significant association. Kaplan-Meier survival curves were produced to report relationships between the risk groups as classified by markers of interest (i.e. single cell invasion and large nuclei).

## Results

Tumor recurrence occurred in 89 (28.6%) of the cases during follow-up. There were 63 (20.3%) patients died of OTSCC, while 95 (30.5%) died of other causes and 153 (49.2%) patients were alive. The median follow-up time was 57.2 months. There were 143 (46%) tumors with single cell invasion, and large nuclei were identified in 141 (45.3%) tumors. Reproducibility between the observers was good in the assessment of single cell invasion (Kappa value = 0.741) and large nuclei (Kappa value = 0.751). The two observers agreed about the presence of single cell invasion and large nuclear diameter in 78% and 86% of the tumors, respectively.

There was an obvious and statistically significant association (*P* < 0.001) between single cell invasion and the presence of large nuclei as most of the tumors presenting with single cell invasion had also large nuclei. In the analyses of the relationship between single cell invasion and clinicopathologic features (Table 1), there was a significant association between single cell invasion and tumor grade (*P* = 0.020). In addition, there was a significant association between the presence of large nuclei and perineural invasion (*P* = 0.020).

**Table 1:** Relationship between single cell invasion, nuclear diameter and clinicopathologic features of early-stage oral tongue cancer

Variable	Single cell invasion		***P*** value	Nuclear diameter		***P*** value
	Absent	Present		Small nuclei	Large nuclei*	
	Number (%) 168 (54%)	Number (%) 143 (46%)		Number (%) 170 (54.7%)	Number (%) 141 (45.3%)	
**Age**			0.189			0.297
≤ 60 years	64 (49.6%)	65 (50.4%)		66 (51.2%)	63 (48.8%)	
> 60 years	104 (57.1%)	78 (42.9%)		104 (57.1%)	78 (42.9%)	
**Gender**			0.378			0.617
Men	93 (56.4%)	72 (43.6%)		88 (53.3%)	77 (46.7%)	
Women	75 (51.4%)	71 (48.6%)		82 (56.2%)	64 (43.8%)	
**TNM Stage**			0.483			0.225
T1N0M0	70 (56.5%)	54 (43.5%)		73 (58.9%)	51 (41.1%)	
T2N0M0	98 (52.4%)	89 (47.6%)		97 (51.9%)	90 (48.1%)	
**Differentiation (Grade)**			**0.020**			0.158
Grade I	62 (59.0%)	43 (41.0%)		56 (53.3%)	49 (46.7%)	
Grade II	76 (58.0%)	55 (42.0%)		79 (60.3%)	52 (39.7%)	
Grade III	30 (40.0%)	45 (60.0%)		35 (46.7%)	40 (53.3%)	
**Perineural invasion**			0.058			**0.020**
No	151 (56.1%)	118 (43.9%)		154 (57.2%)	115 (42.8%)	
Yes	17 (40.5%)	25 (59.5%)		16 (38.1%)	26 (61.9%)	
**Depth of invasion**			0.167			0.049
Superficial (≤ 5 mm)	105 (57.4%)	78 (42.6%)		109 (59.6%)	74 (40.4%)	
Deep (> 5 mm)	63 (49.2%)	65 (50.8%)		61 (47.7%)	67 (52.3%)	
						-
**Nuclear diameter**			**< 0.001**			
Small nuclei	146 (85.9%)	24 (14.1%)		-	-	
Large nuclei	22 (15.6%)	119 (84.4%)		-	-	
**Single cell invasion**			-			**< 0.001**
Absent	-	-		146 (86.9%)	22 (13.1%)	
Present	-	-		24 (16.8%)	119 (83.2%)	

*Large nuclei defined as greater in diameter than 4 nearby small lymphocytes

In survival analyses (Tables 2 and 3), the presence of single cell invasion was significantly associated with cancer-related mortality (i.e., disease-specific survival) both in univariable analysis (HR 2.025 95% CI 1.217–3.368, *P* = 0.007) and in multivariable analysis (HR 2.089, 95% CI 1.224–3.566, *P* = 0.007)). Similarly, single cell invasion was associated with disease-free survival both in univariable analysis (HR 1.647, 95% CI 1.080–2.512, *P* = 0.021) and in multivariable analysis (HR 1.666, 95% CI 1.080–2.571, *P* = 0.021).

**Table 2:** Disease-specific survival analysis of the prognostic significance of single cell invasion, nuclear diameter and clinicopathologic characteristics of early oral tongue cancer (*n* = 311 patients)

Parameter	Univariable Analysis	Multivariable Analysis
	HR (95%CI), ***P*** value	HR (95%CI), ***P*** value
**Age**		
≤ 60 years	Reference	Reference
> 60 years	1.867 (1.096–3.182), *P* = 0.022	2.158 (1.233–3.775), *P* = 0.007
**Gender**		
Male	Reference	Reference
Female	1.215 (0.740–1.992), *P* = 0.441	1.244 (0.738–2.095), *P* = 0.412
**Stage**		
T1N0M0	Reference	Reference
T2N0M0	1.484 (0.866–2.542), *P* = 0.151	1.442 (0.822–2.530), *P* = 0.202
**Differentiation (Grade)**		
Grade I	Reference	Reference
Grade II	1.681 (0.919–3.074), *P* = 0.092	2.094 (1.138–3.851), *P* = 0.018
Grade III	1.580 (0.790–3.159), *P* = 0.196	2.044 (0.996–4.196), *P* = 0.051
**Perineural invasion**		
None	Reference	Reference
Yes	1.269 (0.645–2.495), *P* = 0.490	0.816 (0.403–1.653), *P* = 0.572
**Depth of invasion**		
5 mm or less	Reference	Reference
More than 5 mm	3.042 (1.802–5.137), *P* < 0.001	3.056 (1.779–5.250), *P* < 0.001
**Single cell invasion**		
Absent	Reference	Reference
Present	2.025 (1.217–3.368), *P* = 0.007	2.089 (1.224–3.566), *P* = 0.007
**Nuclear diameter**		
Small	Reference	Reference
Large	1.979 (1.190–3.292), *P* = 0.009	2.070 (1.216–3.523), *P* = 0.007

**Table 3:** Disease-free survival analysis of the prognostic significance of single cell invasion, nuclear diameter and clinicopathologic characteristics of early oral tongue cancer (*n* = 311 patients)

Parameter	Univariable Analysis	Multivariable Analysis
	HR (95%CI), ***P*** value	HR (95%CI), ***P*** value
**Age**		
≤ 60 years	Reference	Reference
> 60 years	1.786 (1.143–2.790), *P* = 0.011	1.948 (1.224–3.101), *P* = 0.005
**Gender**		
Male	Reference	Reference
Female	1.081 (0.711–1.642), *P* = 0.715	0.940 (0.604–1.462), *P* = 0.783
**Stage**		
T1N0M0	Reference	Reference
T2N0M0	0.872 (0.571–1.332), *P* = 0.526	0.831 (0.533–1.296), *P* = 0.415
**Differentiation (Grade)**		
Grade I	Reference	Reference
Grade II	1.111 (0.680–1.816), *P* = 0.673	1.236 (0.752–2.033), *P* = 0.403
Grade III	1.246 (0.717–2.163), *P* = 0.435	1.443 (0.812–2.565), *P* = 0.211
**Perineural invasion**		
None	Reference	Reference
Yes	1.462 (0.839–2.549), *P* = 0.180	1.320 (0.741–2.351), *P* = 0.346
**Depth of invasion**		
5 mm or less	Reference	Reference
More than 5 mm	1.425 (0.938–2.166), *P* = 0.097	1.394 (0.900-2.159), *P* = 0.137
**Single cell invasion**		
Absent	Reference	Reference
Present	1.647 (1.080–2.512), *P* = 0.021	1.666 (1.080–2.571), *P* = 0.021
**Nuclear diameter**		
Small	Reference	Reference
Large	1.641 (1.076–2.503), *P* = 0.021	1.645 (1.067–2.538), *P* = 0.024

Furthermore, the presence of large nuclei (i.e. greater than four small lymphocytes) was associated with cancer-related mortality both in univariable analysis (HR 1.979, 95% CI 1.190–3.292, *P* = 0.009) and in multivariable analysis (HR 2.070, 95% CI 1.216–3.523, *P* = 0.007). In addition, large nuclei were significantly associated with disease-free survival in univariable analysis (HR 1.641, 95% CI 1.076–2.503, *P* = 0.021) as well as in multivariable analysis (HR 1.645, 95% CI 1.067–2.538, *P* = 0.024).

**Fig. 2:**
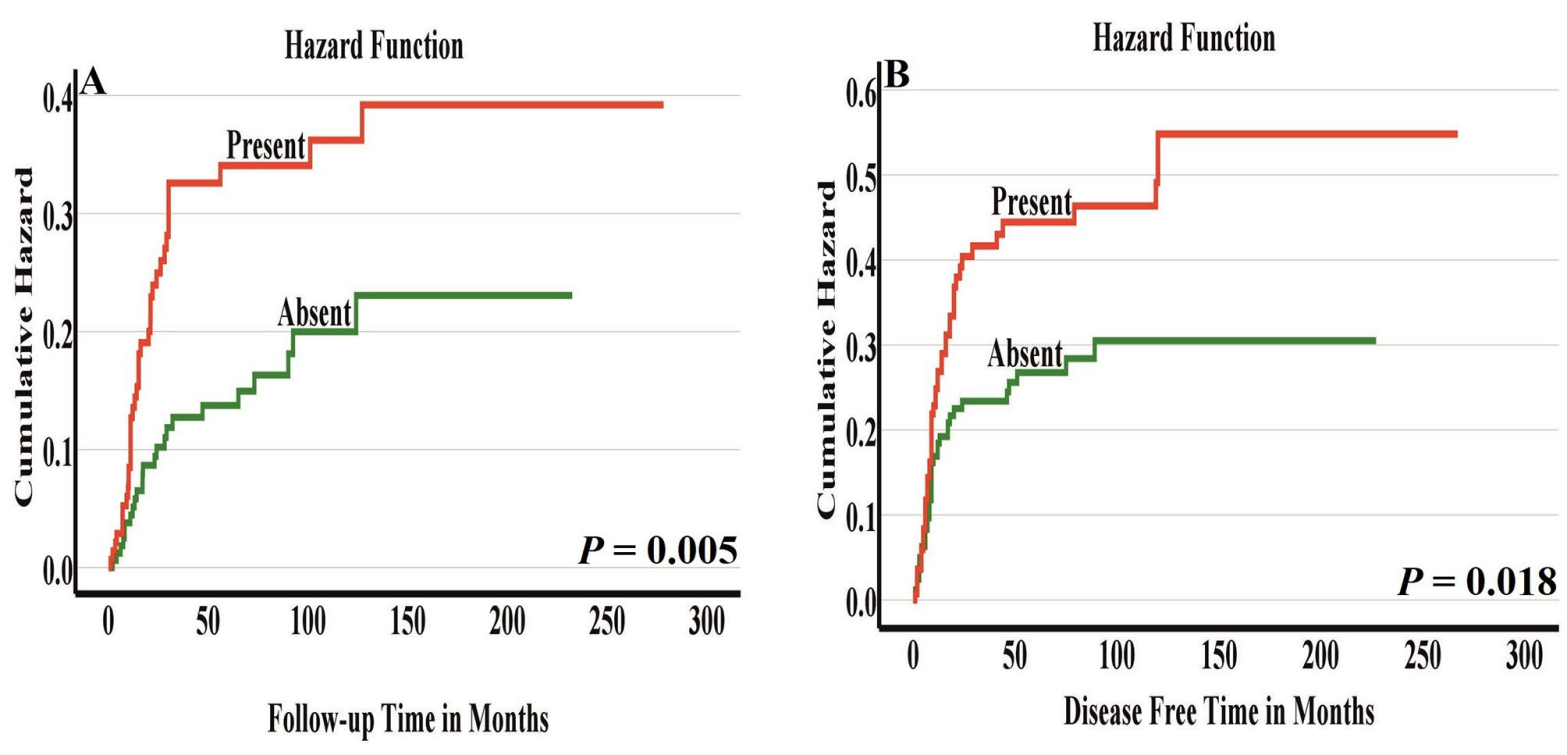
Disease-specific survival **(A)** and disease-free survival **(B)** by single cell invasion. Presence of single cell invasion associated with poorer prognosis

**Fig. 3:**
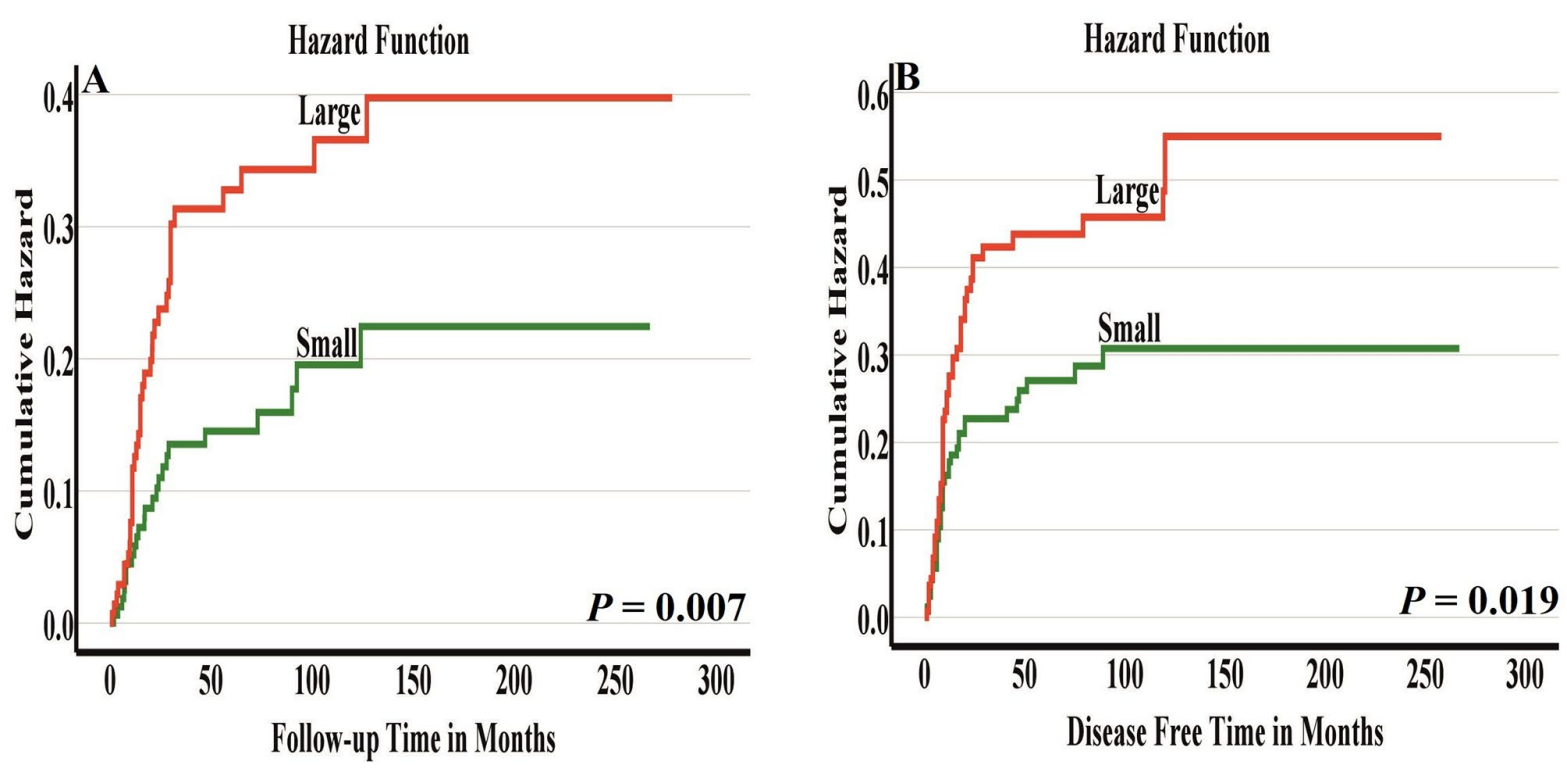
Disease-specific survival **(A)** and disease-free survival **(B)** by nuclear diameter. Cases having large nuclei associated with worse survival

To ensure the independence of single cell invasion and large nuclei as prognostic parameters, routinely considered prognostic factors including tumor stage, tumor grade, perineural invasion, depth of invasion and patient age and gender were included in our multivariable analyses (Tables 2 and 3). Furthermore, Kaplan-Meier curves clearly showed the prognostic significance of single cell invasion (Fig. 2A-B) and large nuclei (Fig. 3A-B) in dividing the cases into low-risk and high-risk groups.

## Discussion

The clinical behavior of early-stage OTSCC is unpredictable in many cases in which routinely used classifier/s (i.e. TNM stage or WHO grade) fail in predicting the risk of recurrence and/or risk of cancer-related mortality at the time of treatment planning [20, 21]. This makes the identification of high-risk early OTSCC patients one of the key challenges in daily practice. Thus, there is a need to identify promising histopathologic classifiers that can aid in accurate prediction of tumor behavior in OTSCC at an early stage. In recent years, research attempts have been made to introduce improved histopathologic markers (and grading systems of multiple markers) for evaluation of morphological, cellular, and/or nuclear features assessed in HE-stained Sections. [22, 23]. Such markers are cost-effective and can provide histologic prognostication beyond the routinely reported WHO grading system. Thus, they can supplement the TNM staging system and improve clinical decision making. In the present study, for the first time we report on prognostic significance of single cell invasion and large nuclei (i.e. greater that four lymphocytes) in a large multicenter cohort of early OTSCC. Both of these morphological features were associated significantly with survival in early OTSCC.

In many studies, the influence of single cell invasion [11, 12] and variations in nuclear morphological features [18, 19, 24, 25] have been associated with prognosis of different tumors. Similarly, single cell invasion was associated with aggressive tumor behavior in the current study (Tables 1, 2 and 3) and in previous studies [11, 12]. In the same vein, our current study found a significant relationship between enlarged nuclei (i.e. being more than the sum of diameters of four small nearby lymphocytes) and aggressive clinical behavior including a high risk for recurrence and cancer-related mortality (Tables 1, 2 and 3). Similar finding was reported in previous studies of other cancers [17, 26]. Reasons for the aggressive behavior of tumors with large nuclei are likely manyfold. It has been assumed that an increase in the number of chromosomes in cancer cells is associated with an increase in nuclear size [15]. Furthermore, aneuploidy, which is associated with poor survival in OTSCC, may also contribute to the increase of nuclear size [15]. For single cell invasion, dissociation and migration of individual cell/s has been observed [27]. Of note, single cell invasion has been linked to epithelial-mesenchymal transition (EMT) and those single cells might express characteristics of cancer stem cells [28]. It is well-documented that EMT has shown an association with cancer cell invasion and progression of the tumor [29]. In addition, loss of cytokeratin and gain of vimentin expression indicate aggressive tumor behavior [30]. Furthermore, cytokeratins have been suggested to play a crucial role during the early stages of EMT, serving as a priming step for the induction of EMT in the epithelial cells [31]. Therefore, cytokeratin and EMT immunostaining markers could aid in the understanding of the biological background of single cell invasion.

Importantly, we demonstrate good reproducibility between the observers when evaluating single cell invasion and nuclear diameter. This suggests that these two parameters should be considered for routine practice of pathologists after further validation. Furthermore, the use of machine learning classifiers for the evaluation of various cellular/nuclear characteristics has already been successfully introduced for other tumor types [16, 19, 32]. Such an automated method could facilitate the assessment of single cell invasion and large nuclei also in OTSCC. Remarkably, we found that both single cell invasion and large nuclei were independent prognostic markers after we adjusted the multivariable analysis with the classical prognostic parameters including tumor stage, WHO grade, perineural invasion and depth of invasion. This indicates that single cell invasion and large nuclei can add value to risk stratification in early OTSCC beyond the classic prognostic parameters that are used currently in daily practice.

There are some limitations in our study that need to be mentioned. First, the study is retrospective in nature, and therefore prospective studies are necessary to validate our findings. Second, the patients were from different time periods. In addition, postoperative treatment of the patients, as well as the management of recurrent and metastatic tumors was not analyzed. Furthermore, immunostaining for cytokeratin or EMT markers could not be performed for this study, as additional sections from the various hospitals were not available. Moreover, no analytic software in oral tongue cancer is presently available for the assessment of single cell invasion and nuclear diameter. The development and training of an artificial intelligence (AI) neural networks for the assessment of the two markers would require two large cohorts (one for training and another for validation), which were not available for the current study. Development of AI systems for the assessment of the present markers in oral cancer remains important in future research.

## Conclusions

We shed light on the significance of single cell invasion and large nuclei in prognostication of early OTSCC. Our findings suggest that both can be used to select high-risk oral tongue cancer patients who should be considered for multimodality treatments, even if diagnosed at an early stage. As our histopathologic evaluation was conducted using HE-stained sections available in routine daily practice, the findings of our study can be easily validated in different cohorts and this should be undertaken in future research.

## Data Availability

Data used in this study is available from the corresponding author upon a reasonable request.

## Abbreviations

CI: Confidence interval
DFS: Disease-free survival
DSS: Disease-specific survival
HE: Hematoxylin and eosin
HR: Hazard ratio
OTSCC: Oral tongue squamous cell carcinoma
WHO: World Health Organization
